# 2349. Natural Killer Cells and BNT162b2 mRNA Vaccine Reactogenicity and Durability

**DOI:** 10.1093/ofid/ofad500.1971

**Published:** 2023-11-27

**Authors:** Elizabeth K Graydon, Tonia Conner, Kim Dunham, Cara Olsen, Emilie Goguet, Allison Segard, Si’Ana A Coggins, Marana S Tso, Emily C Samuels, Belinda Jackson-Thompson, Matthew S Moser, Alyssa R Lindrose, Monique Hollis-Perry, Gregory Wang, Santina Maiolatesi, Yolanda Alcorta, Anatalio Reyes, Mimi Wong, Kathy Ramsey, Julian Davies, Edward Parmelee, Orlando Ortega, Margaret Sanchez Edwards, Sydney Moller, Jon Inglefield, David Tribble, Timothy Burgess, Robert O’Connell, Allison M Malloy, Simon Pollett, Christopher Broder, Eric D Laing, Stephen Anderson, Edward Mitre

**Affiliations:** HJF; Uniformed Services University, Bethesda, Maryland; Uniformed Services University, Bethesda, Maryland; Frederick National Laboratory for Cancer Research, Frederick, Maryland; Uniformed Services University, Bethesda, Maryland; HJF, USUHS, Bethesda, Maryland; Uniformed Services University, Bethesda, Maryland; HJF/USUHS, Rockville, Maryland; HJF, USUHS, Bethesda, Maryland; HJF, USUHS, Bethesda, Maryland; Uniformed Services University, Bethesda, Maryland; HJF, USUHS, Bethesda, Maryland; HJF, USUHS, Bethesda, Maryland; Clinical Trials Center, Infectious Diseases Directorate, NMRC, Silver Spring, Maryland; Naval Medical Research Center, Bethesda, Maryland; HJF, CTC NMRC, Silver Spring, Maryland; CTC, NMRC, General Dynamics Information Technology, Bethesda, Maryland; CTC, NMRC, General Dynamics Information Technology, Bethesda, Maryland; CTC, NMRC, General Dynamics Information Technology, Bethesda, Maryland; General Dynamics Information Technology; Clinical Trials Center, Infectious Diseases Directorate, NMRC, Silver Spring, Maryland; Infectious Diseases Clinical Research Program, Henry M. Jackson Foundation, Bethesda, Maryland; Infectious Disease Clinical Research Program, Department of Preventive Medicine and Biostatistics, Uniformed Services University of the Health Sciences, Bethesda, Maryland; HJF, Infectious Diseases Clinical Research Program, Bethesda, Maryland; Infectious Disease Clinical Research Program, Department of Preventive Medicine and Biostatistics, Uniformed Services University of the Health SciencesHenry M. Jackson Foundation for the Advancement of Military Medicine, Bethesda, Maryland; Frederick National Laboratory for Cancer Research, Frederick, Maryland; Frederick National Laboratory for Cancer Research, Frederick, Maryland; Infectious Disease Clinical Research Program, Department of Preventive Medicine and Biostatistics, Uniformed Services University of the Health Sciences, Bethesda, MD, USA, Bethesda, Maryland; Infectious Disease Clinical Research Program, Department of Preventive Medicine and Biostatistics, Uniformed Services University of the Health Sciences, Bethesda, MD, USA, Bethesda, Maryland; Infectious Disease Clinical Research Program, USUHS, Bethesda, Maryland; Department of Pediatrics, Uniformed Services University of the Health Sciences, Bethesda, MD, USA, Bethesda, Maryland; Infectious Disease Clinical Research Program, Department of Preventive Medicine and Biostatistics, Uniformed Services University of the Health Sciences, Bethesda, MD, USA, Bethesda, Maryland; Uniformed Services University, Bethesda, Maryland; Department of Microbiology and Immunology, Uniformed Services University, Bethesda, MD, Bethesda, Maryland; Basic Science Program, Frederick National Laboratory for Cancer Research, Frederick, Maryland; Uniformed Services University, Bethesda, Maryland

## Abstract

**Background:**

Natural killer (NK) cells can both amplify and diminish immune responses to vaccination. Studies in humans and animals have observed NK cell activation within days after mRNA vaccination. In this study, we sought to determine if baseline NK cell frequencies, phenotype, or function correlate with antibody responses or inflammatory side effects induced by the Pfizer-BioNTech COVID-19 vaccine (BNT162b2).

**Methods:**

We analyzed serum and peripheral blood mononuclear cells (PBMCs) from 188 participants in the Prospective Assessment of SARS-CoV-2 Seroconversion study, an observational study evaluating immune responses in healthcare workers. Baseline serum samples and PBMCs were collected from all participants prior to any SARS-CoV-2 infection or vaccination. Spike-specific IgG antibodies were quantified at one and six months post-vaccination by microsphere-based multiplex immunoassay. NK cell frequencies and phenotypes were assessed on pre-vaccination PBMCs from all participants by multi-color flow cytometry, and on a subset of participants at time points after the 1^st^ and 2^nd^ doses of BNT162b2. Inflammatory side effects were assessed by structured symptom questionnaires, and baseline NK cell functionality was quantified by an *in vitro* killing assay on participants that reported high or low post-vaccination symptom scores.

**Results:**

Key observations include: 1) circulating NK cells exhibit an increase in CD56dim CD16- NK cells compared to baseline levels, providing evidence of NK cell activation in the week following vaccination, 2) individuals with high symptom scores after 1^st^ vaccination had higher pre-vaccination NK cytotoxicity indices compared to individuals with low symptoms scores (195.7 [SD 170.1] vs 118.5 [SD 77.5], p=0.04), and 3) pre-vaccination NK cell numbers were negatively correlated with spike-specific IgG levels six months after two BNT162b2 doses (Rho= -0.14, p=0.043).

**Conclusion:**

These results suggest that NK cell activation by BNT162b2 vaccination may contribute to vaccine-induced inflammatory symptoms and reduce durability of vaccine-induced antibody responses.

**Disclosures:**

**David Tribble, MD, DrPH**, AstraZeneca: The IDCRP and HJF were funded to conduct an unrelated phase III COVID-19 monoclonal antibody immunoprophylaxis trial as part of US Govt COVID response **Timothy Burgess, MD, MPH**, AstraZeneca: The IDCRP and the Henry M. Jackson Foundation (HJF) were funded to conduct an unrelated phase III COVID-19 monoclonal antibody immunoprophylaxis trial **Simon Pollett, MBBS**, AstraZeneca: The IDCRP and the Henry M. Jackson Foundation (HJF) were funded to conduct an unrelated phase III COVID-19 monoclonal antibody immunoprophylaxis trial

